# Exploring nurses’ experience with benzathine penicillin administration for patients with rheumatic heart disease in Ethiopia: a qualitative study

**DOI:** 10.1186/s12912-026-05197-4

**Published:** 2026-08-07

**Authors:** Habtamu Abera Areri, Henok Tadele, Sosina Workineh, Senbeta Guteta, Abebe Gebremariam, Elizabeth C. Rhodes, Leslie Johnson, Saria Hassan, Rebecca Gary

**Affiliations:** 1 School of Nursing and Midwifery, College of Health Sciences, Addis Ababa University, Addis Ababa, Ethiopia; 2 Department of Pediatrics and Child Health, School of Medicine, College of Health Sciences, Addis Ababa University, Addis Ababa, Ethiopia; 3 Department of Internal Medicine, School of Medicine, College of Health Sciences, Addis Ababa University, Addis Ababa, Ethiopia; 4 Nell Hodgson Woodruff School of Nursing, Emory University, Atlanta, GA USA; 5 Hubert Department of Global Health, Rollins School of Public Health, Emory University, Atlanta, GA USA; 6 School of Medicine, Emory University, Atlanta, GA USA

**Keywords:** Nurse, Benzathine penicillin, Rheumatic fever, Rheumatic recurrence, Rheumatic heart disease, Barriers, Facilitators

## Abstract

**Background:**

Nurses have a unique opportunity to lower rheumatic fever recurrences through the administration of Benzathine Penicillin G (BPG) a widely used secondary prophylaxis. However, fear of side effects, lack of administration skills, and communication among nurses has had a negative impact on BPG administration in Africa. Overall, the administration of BPG remains low. Therefore, this study aims to understand the experience of nurses with BPG administration in Tikur Anbessa Specialized Hospital to inform an intervention to improve BPG administration rates in Ethiopia.

**Methods:**

This qualitative research was guided by capability, opportunity, motivation-Behavior model (COM-B) and the Behavior change Wheel (BCW). In-depth interviews were conducted among cardiac and generic practice nurses with BPG administration experiences from adult and pediatric cardiac service areas. Thematic analysis was carried out using Braun and Clark’s approach.

**Results:**

The study included 26 both general and cardiac nurses. Capability barriers commonly discussed were limited skills in safe BPG injection and misunderstanding of BPG prophylaxis. Lack of reference guidelines, unavailability of BPG and other medications, limited healthcare resources (staff, supplies, injection room) and limited inter-professional communication were identified as opportunity barriers. Demotivators of BPG administration had limited intention to provide BPG due to fear of anaphylaxis, legal accountability and lack of injection administration confidence. However, working experience with RHD patients, knowledge of RHD, availability of needed resources, support from nurse colleagues, absence of side effects during previous injections and professional responsibility were identified as enablers of BPG administration.

**Conclusions:**

Barriers of BPG administration such as poor nurses’ intention due to fear of adverse effects, medico-legal concern, and absence of consent and injection room were mapped onto COM-B and the BCW were used to identify potential future interventions. Training, education, environmental restructuring and enablement of what were commonly identified interventions to enhance nurses’ BPG administration. Multicenter studies are recommended to better understand the factors and address barriers. A study is recommended to test effectiveness of interventions recommended based on the research findings.

**Clinical trial number:**

Not applicable.

**Supplementary Information:**

The online version contains supplementary material available at https://doi.org/10.1186/s12912-026-05197-4.

## Introduction

Intramuscular Benzathine penicillin G (BPG) is the major cornerstone for secondary Rheumatic Heart Disease (RHD) prophylaxis [1, 2]. To prevent the progression of RHD through rheumatic fever recurrence, intramuscular BPG is recommended to be given every three or four weeks [1–4]. However, BPG injections have been linked to severe pain, regional muscle tension, and discomfort [5]. Fatal events have also been documented among severe RHD patients who received BPG injections, with the main cause of death being compromised cardiac reserve and not anaphylaxis [6, 7]. Another review also documented the rarity of sudden death following BPG injection and suggested an alternate mechanism of medication administration to reduce the risk for sudden death [1]. The American Heart Association has provided advice on oral prophylaxis over IM BPG in RHD patients with severe valvular lesions and also advocates oral prophylaxis over IM BPG in RHD patients with severe valvular lesions with limited cardiovascular reserve [2].

A study that focused on 30 healthcare providers from 14 African nations details difficulties in administering BPG. According to this report, the primary obstacles in Africa were the high rates of BPG unavailability, the absence of BPG administration guidelines, the lack of emergency kits to treat anaphylactic reactions, and the lack of confidence among medical professionals to treat BPG allergies. Pain from BPG injection and local muscle tension was reported to be a reason for reluctance to provide BPG injection among providers [4]. It has been shown that nurses are not well-versed in conversational strategies when administering injections which has been shown to lessen injection pain by 25% [8]. In Zambia, the educational and access-to-medicine program showed an increase in the appropriate use of BPG for the prevention and management of RHD. Appropriate education of healthcare personnel, together with confidence building around the management of allergic drug reactions, followed by several low-cost ongoing supportive measures have the potential to significantly improve rates of BPG delivery [9]. Another Ugandan study documented barriers to BPG which included poor patient-provider communication and poor availability of clinics and providers trained to administer injections [10]. Barriers identified in a study conducted in Sudan, were lack of faith in local healthcare systems, poor ancillary services, and patient lack of understanding of RHD. RHD patients who did not understand their condition could worsen due to limited utilization of BPG prophylaxis [11].

By being the most closely involved in many aspects of patient care, nurses have a remarkable and unique opportunity to assist with lowering rheumatic fever recurrences among RHD patients. An absence of skills, trust and communication among nurses negatively influences BPG administration [12]. The nurse’s attitude towards BPG also matters in establishing a rapport with patients in the process of treatment. Raising healthcare professionals’ knowledge of RHD prophylaxis is essential to enhancing RHD disease progression control. Education focusing on BPG prophylaxis is crucial to reducing misunderstanding and improving awareness of both nurses and RHD patients [12]. It is also recommended that BPG should be administered by trained nurses [1].Thus, nurses need ongoing education, skills training, and professional development on BPG administration and its importance to ensure adequate BPG management and adherence to evidence based guidelines [12–14].

The delivery rate of BPG for RHD prophylaxis has been found to be low in Ethiopia [8, 15, 16]. Fear of BPG allergic reactions was one of the main reasons for the reluctance to administer BPG. A negative attitude toward the use of BPG as a secondary prophylaxis for RHD was also documented among junior healthcare professionals [8, 16]. Besides, in recent study, lack of volunteer BPG providers and providers’ fear of the medication, remain key challenges for RHD patients to get their monthly prophylaxis [16]. Nurses are key front-line clinicians that provide most of the patient BPG injections in Ethiopia and therefore are central to prevention of RHD progression. According to an Ethiopian study on nurses’ awareness of BPG prophylaxis and RHD, only 48.7% of nurses were informed about RHD during their training. Receiving in-service training on RHD and having more work experience in cardiac centers were linked to better knowledge of RHD care [15]. Nurses have cited the potential for needle blockage during BPG administration and a lack of protocols for administering medication as the primary causes of BPG administration challenges [8]. Moreover, little research has been conducted to investigate the challenges nurses face when giving BPG injections to RHD patients. Despite this information there remains a gap in BPG administration. This study aims to understand the experience of nurses with BPG administration to inform an intervention to improve BPG administration rates in Ethiopia.

## Methods

### Conceptual framework

The Capability, Opportunity, Motivation and Behavior (COM-B) model was chosen because it is thorough and adaptable enough to analyze any behavior in any situation. The COM-B model proposes an individual’s capability (physical such as skills and psychological such as knowledge), opportunity (physical such as availability of medications and social such as social support) and motivation (reflective such as intentions and automatic such as emotions) are necessary requirements for any behavior to occur. This model has been widely used to explore barriers and facilitators to healthcare providers’ practice [17–19]. Furthermore, the COM-B model is a part of the Behavior Change Wheel (BCW), an intervention development framework that connects the COM-B components and aids in the selection of intervention strategies and techniques that are likely to successfully address the barriers of the proposed behavior [20, 21].

### Study setting

The study was conducted in Tikur Anbessa Specialized Hospital, which is located in the heart of Addis Ababa, the capital city of Ethiopia, and is under the College of Health Sciences of Addis Ababa University. It is the national referral hospital where several cardiac/RHD interventions are provided. There are over 10,000 RHD patients in chronic care follow-up in this hospital and most of them are on a lengthy waiting list for definitive surgical or device-related interventions. RHD follow up care and treatment in this hospital is carried out by cardiologists and cardiac nurses in most cases. Currently, according to the human resources of the college, 765 nurses were working in the hospital in different units.

### Study design

A descriptive qualitative design was utilized. This study design was applied to enable deeper exploration and a better understanding of nurses’ experience of BPG administration. The Consolidated criteria for Reporting Qualitative Research( COREQ) reporting format [32-item checklist) was followed to enhance methodological rigor and transparency of the research findings [22].

### Sampling and recruitment

The study was conducted with cardiac and general nurses (*n* = 20–30) who worked with RHD patients at Tikur Anbessa Specialized Hospital from July to September 2024. A purposive sampling was used to recruit study participants from adult and pediatric cardiac care centers of the hospital to achieve maximum variation among study participants. Variability of study participants is assumed by including different categories of nurses from different units such as adults and pediatrics cardiac follow up clinics, injection rooms, and cardiac in patients. The inclusion criteria were nurses with BPG administration experience or cardiology specialty graduate nurses or general practice nurses. Nurses with a minimum of six months’ experience in BPG administration were included. To recruit nurses, a call was posted in their workplace in addition to personal communications, and finally we received 37 interested nurses for participation in our study. The researchers then evaluated all interested parties for eligibility before setting up the interview schedule. Interviews were then conducted until the data saturation was reached in private room The interview was conducted in absence third party in the interview environment.

### Interview guide and data collection

The data collection interview guide with nurses was created using a COM-B model conceptual framework [20, 21]. Semi-structured interview questions that examined nurses’ demographics and BPG administration practices were included in the interview guide (Supplement Table 1]. The interview guide was translated into the local language in consultation with language expert and checked for consistency and accuracy of translation by translating back to original language. The guide was a pilot tested on five nurses and evaluated for clarity, content and time. Prior contact with purposively selected participants was made to arrange time of interview and for a brief discussion of purpose of the study. Two researchers (HA & SW) with training in qualitative research conducted in-depth interviews. HA is a PhD holder in nursing with extensive experience of qualitative research approach, cardiovascular research, nursing practice and guided the whole process of interview and data analysis. The interview took 30–60 min. To prevent distractions and preserve privacy, all interviews were held in a private room following a brief description of the study’s goal and the acquisition of informed permission. All interviews were audio recorded, transcribed, and translated into English before the final analysis. Transcripts were sent to the participants to seek their feedback and verify whether the interpretations accurately reflected their perspectives.

### Trustworthiness

Trustworthiness of the study was ensured through varies measures taken. Credibility verified through audio-recorded and transcribed verbatim. Two independent investigators independently coded, analyzed and discussed the process when there were differences. Transferability was ensured through detailed description of the study area, participants characteristics, research design, data collection, analysis process and themes emerged. Dependability ensured by carefully documenting each stage of the research process, including interview transcripts, the coding process and key decisions made from data collection to analysis. Conformability was verified by using field notes and audio records for an audit trial to confirm that the data interpretation accurately reflects the participants’ own words rather than the researchers’ opinions or views by using the participants’ own words from interview transcripts. Additionally, a reflexive journal was kept documenting the researcher’s reflections, assumptions, and decisions.

### Data analysis

All transcripts were imported in *ATLAS ti.*, software, and the thematic content analysis method developed by Braun and Clark’s was followed for the final analysis. The data was coded by two independents researchers (HA and SW), who then compared the codes for consistency and discussed any discrepancies. Each theme and sub-theme are described using illustrative quotes. The responses from each participant were sequentially coded, with participant 1 being designated as N1. One of the researchers (HA) has extensive training and experience in using qualitative research. In addition, HA has had experience using the COM-B model. Thus, the emergent themes were grouped by the COM-B constructs/domains [20, 21]. Six constructs and the three primary COM-B domains made up the mapped themes.

### Ethics approval and consent to participate

To conform with the Declaration of Helsinki (1964) and Population Screening Act, ethical approval for the study was obtained from Addis Ababa University, College of Health Sciences institutional research Ethics Review Committee (024/24/pedi). Permission was obtained from the hospital management. Participation was determined by the person without any external influence to participate or not. Written informed consent was obtained from all individual participants. Participants of this study had the right to withdraw anytime from the research project without any negative consequences. Information was collected in private rooms and always kept confidential and secure.

## Results

### Participants characteristics

Based on data saturation, 26 nurse interviews were included in the study. Many (*n* = 15, 58%), of the participants were female. Their ages ranged from 25 to 55 years and the mean number of years working was 10.12 (range: 3 to 27 years). Majority of participants (73%) had a bachelor’s degree, while the remaining participants (*n* = 7)] were masters prepared in cardiovascular nursing. All participants had direct practical experience in RHD care. Most of the nurses included (*n* = 21) had over a year working experience BPG administration experience and the remaining had six to a year experience. The study participants were coded from N1 to N26.

### Thematic analysis

All barriers and facilitators of BPG administration were mapped to the domains of the COM-B model. Below, the findings were further explained in relation to the six COM-B model domains (Table 1).

**Table 1:** Barriers and facilitators of nurses’ BPG administration

Themes		Sub-themes	
COM-B Domains		Facilitators	Barriers
Capability	Physical	Working with RHD patients	Limited skills to manage complications
			Lack of training in safe injection methods
	Psychological	Knowledge of RHD	Medication misunderstanding and lack of awareness of BPG prophylaxis guidelines
Opportunity	Physical	Availability of resources	Lack of reference guidelines
		Capacity building program	Medication unavailability
		Site/location where injections are administered	Limited Healthcare resources: staff, supplies, injection room
	Social	Support from colleagues	Rejection by RHD patients or community
			Limited communication and inter-professional collaboration
Motivation	Reflective	Professional commitment to BPG administration	Limited intention to provide
			Intention vs. consequence
			Limited belief in Capability/Lack of confidence
	Automatic	Absence of reactions during previous injection	Fear of shock/collapse or other side effects
			Fear of accountability
			Absence of consent

### Psychological capability facilitators

#### Knowledge of RHD

A significant number of nurses interviewed appear to understand the importance of BPG in halting the progression of RHD. The following are other comments made by participants regarding BPG prophylaxis in halting the progression of the disease: “*I am aware that this medication is crucial for RHD patients to avoid complications and deterioration.” [N2].* Another nurse also added: “*BPG is important in preventing or slowing the progression.” (N11).* Additionally, participants in this study briefly discussed how having a thorough understanding of BPG prophylaxis and its anticipated side effects could enhance the tendency of BPG administration for patients in need of medication. Nurses described their understanding of BPG and its related management: “*When you know the benefit of the medication*,* you do have a high tendency of administering the injection.* [N12]. It was also spoken: “*Knowing the side effects of BPG ahead of its administration makes you prepared for any outcomes and improves your tendency of administering the injection.” [N20].* Another respondent mentioned how his degree aided him in managing prophylaxis of BPG. Pursuing a master’s degree in cardiovascular nursing was said to facilitate the delivery of BPG prophylaxis. [N26].

### Physical capability facilitators

#### Working with RHD patients

Most of the participants spoke about how working with RHD patients or at cardiac clinics gave them the chance to comprehend the nature of the condition and gave them the confidence to give BPG to patients with RHD. The significance of working with patients who have RHD was emphasized by one of the cardiovascular nurses as follows:

I have worked in cardiac care for a total of 16 years. My seven-year experience was directly related to working with RHD patients. They are young and sometimes they face difficulty in refilling their medication (she feels emotional). Anyway, it is touching, and I am happy to work here to contribute to my side. The condition of my patients touches me a lot and motivates me more to support them. [N1].

Nearly every nurse mentioned that they had recently administered BPG. Positive experience significantly increased the interest of nurses in administering BPG. Respondents emphasized the following aspects of their BPG administration experience:

My extensive experience of administering BPG in both private and public hospital settings has given me a positive feeling about medication. Except for hearing here and there rumors about its side effects, I have not seen any complications in my life. Now I have no reason for not providing the medication. [N3].

According to respondents, having the chance to interact with nurses coming from abroad and gain experience administering BPG while working in cardiac care units enabled them to handle the administration of BPG prophylaxis and associate complication management with confidence. Many cardiac nurses were enrolled in specialized training, and they were completely confident in their ability to administer BPG prophylaxis. One reply emphasized the significance of shared experience in the following ways: “*I have got experience sharing opportunities from cardiac professionals coming from abroad which helped me a lot in dealing with BPG medication.”[N26].*

### Physical opportunity facilitators

#### Availability of resources

Most nurses concurred that having access to supplies such as medications, syringes, comfortable injecting rooms, and a well-resourced BPG administration team would facilitate their BPG delivery. Before beginning the administration, they all talked about ensuring the availability of all the necessary materials that could facilitate their BPG administration practice. Most nurses who participated in this study, for instance, agreed that using two needles or syringes could lessen medication blockage during preparation and injection. One syringe or needle will be used to prepare the drug, and the other will be used to provide the injection because this medication clots quickly and makes injection challenging. [N6, N9]. Nearly all nurses who participated in the interview emphasized how essential it is to ensure the availability of medications lidocaine/diclofenac and adrenaline before the administration of BPG. Nurses also talked about how vital it is to guarantee that necessary medications are available: “*It is crucial to collect emergency drugs (such as adrenaline)*,* and painkillers (such as lidocaine or diclofenac) prior to giving the medication.” [N2].*The nurses next discussed the importance of staff preparation for accurate task completion. Additionally, nurses discussed the significance of emotionally and physically preparing RHD patients. In one of the responses, preparation required to allow for the safe administration of BPG was stated as:

Complete the patient and medication preparations, then work with other team members. In order to avoid shock, the patient should be in a lying position. [N9].

#### BPG injection site/location

A significant number of nurses stressed that the site of injection has an influence on BPG administration practice. It is agreed that RHD patients better get BPG injections with minimal resistance if given at a big hospital level with better emergency care facilities. “*Because hospitals have better capacity and equipment*,* I believe that BPG should be administered at the hospital level. Compared to health centers*,* hospitals have greater staff and equipment.”[N17].* It is assumed that in the event of a side effect, the patient will be easily managed at a more advanced medical facility. Another nurse indicated the following as the things she believed would make administering BPG more convenient:

It would be better if it could be given anywhere in any healthcare facility with adequately equipped emergency care providers and supplies. Because, if a patient gets into shock or unexpected side effects happen, you can find emergency care professionals/doctors and emergency medication as soon as possible. So, administering BPG injections in such a facility would improve nurses’ intention to provide the medication.”[N19].

Overall, injection sites in better healthcare facilities were associated with better nurses’ intention of BPG administration. Most of the nurses agreed that RHD patients better get BPG injections in hospitals with better emergency care facilities.

### Social opportunity facilitators

#### Support from colleagues

Many nurses concurred that having other staff members present in the injection room while administering a BPG injection reduces anxiety and boosts self-assurance. “*If you are the only nurse in the injection room*,* you may lose confidence to administer the injection.” [N7].* Staff availability during BPG administration enhances the procedure for several reasons, as explained:

It is preferable to administer the injection alongside other personnel. Because, if something gets to happen, you will manage it together. When one nurse starts giving emergency medication, the other goes to call an emergency doctor. So, the increased number of staff during BPG administration is a positive thing. [N19].

It was also added that the presence of adequate staff in the injection room improves *service quality and reduces patient waiting times*. *[N12*,* N17].* Nurses were also encouraged to administer BPG by other social considerations, such as being accepted by RHD patients. Nurses highlighted “*getting accepted”* by their patients encouraged them to administer BPG prophylaxis most of the time. [N6, N11, N14].

### Reflective motivation facilitators

#### Professional commitment

The study indicated that those nurses who understood their professional commitment were in favor of BPG administration. One of the nurses stated his role as:

One of my professional duties is administering BPG injections. [N9M].

Another male study participant also discussed his professional commitment as: *“I am fully committed to administering the medication to my patients.” [N11].* It was discussed that a significant number of nurses firmly believe in helping patients with RHD to take their regular monthly medications.

### Automatic motivation facilitators

#### Absence of reaction during previous injections

It was recognized that nothing prevents nurses who have never encountered the negative effects of BPG from administering the medication. One response claims that the absence of side effects is still the main reason he continues to administer BPG, and it reads as follows: “*Since I haven’t had any clotting or adverse effects*,* I don’t hesitate to administer BPG.” [N20].*

Another nurse added that she had no side effects or complications of BPG in her professional career and discussed her motivation to provide BPG as:

I do not have negative feelings toward this drug, and nothing prevents me from providing benzathine penicillin for RHD patients. [N3].

### Physical Capability barriers

#### Limited skill in complication management

Most of the time, nurses were unable to administer BPG due to their inadequate ability to manage complications. Respondents typically gave the following explanation for why they don’t give BPG injections:

I can’t administer BPG if I don’t know what to do when the shock occurs. [N15].

It was frustrating to provide BPG especially when you feel incompetent of managing its complication. In many cases, I feel uncomfortable when it comes to managing its complications. [N26].

From the discussion, it feels like many of the nurses said that they had some limitations around complication management.

#### Lack of training on BPG injections

All nurses agreed that training is critically important for safe BPG administration. Only cardiac nurses could get training on BPG and related matters. The remaining nurses in the study discussed how lack of training on safe injections affected their BPG administration. “*Training does help you become more confident when giving the injection and get rid of your apprehension. However*,* there is no training on BPG administration and related management.” [N23].* Another female nurse added: *“To reduce stress*,* we all need to update our knowledge and abilities.” [N19].* To avoid legal repercussions, nurses must also receive training on how to administer BPG injections safely. The administration of BPG and its associated repercussions continue to raise significant medicolegal concerns. Nurses talked about how their rights and responsibilities are made clear through training. Respondents in this study also talked about nurses’ legal protections if BPG adverse effects occur. Majority of the nurses discussed the significance of receiving instruction on safe BPG injection practices and associated medico-legal matters.

According to nurses, BPG management is now based on their own experiences. Every medical professional is aware of BPG prophylaxis. Nonetheless, it is still difficult to administer BPG practically and deal with unforeseen adverse effects. In addition, nurses’ attitudes toward BPG administration are adversely affected by a lack of effective training regarding safe BPG injection, complication treatment, and rumors regarding BPG prophylaxis. Although nurses understand the significance of BPG, they are not proficient in its administration or associated treatment. [N8, N13, N20, N12]. Additionally, a nurse describes the lack of training: “*Positive attitude development and a reduction in nurses’ apprehension are two benefits of training. The training opportunity*,* however*,* is either nonexistent or very limited.” [N14].*

### Psychological capability barriers

#### Medication misunderstanding and lack of awareness of BPG prophylaxis guidelines

Majority of the nurses who took part in the study stated that the main reason BPG was not given was because of misunderstanding about this medicine. Some nurses had misconceptions regarding BPG prophylaxis, claiming that BPG is not the recommended medication because of its adverse effects and has been taken off the market. According to a nurse’s response: “*…… BPG has been halted from the market and don’t know if it can be still prescribed for RHD patients as prophylaxis.” [N21].* On the other hand, awareness of the side effects of BPG prevented nurses from providing BPG injections to their RHD patients.

Awareness or knowing the side effects of the medication prevents the nurse from providing the injection in the absence of needed equipment especially if the patient is new to the medication [BPG]. [N17].

Additionally, nurses were unaware that there were guidelines for administering BPG. *“I am not aware of availability guidelines on BPG injection*,*”* stated in one of the replies. [N9]. Another nurse gave the following explanation of how he understood the existence of guidelines:

I’m not sure if this hospital has any protocols for managing BPG. [N11].

### Physical opportunity barriers

#### Unavailability of medication

The inaccessibility of BPG and other emergency medications continues to be a major barrier to BPG delivery in nearly all of the included interviews. Nurses emphasized that the delivery of BPG have significantly impacted unavailability of emergency medication. [N10, N14]. Overall, the administration of BPG prophylaxis was impacted by the healthcare system’s inadequate ability to guarantee the availability of BPG and other necessary medications. Medication unavailability was described as follows in other interviews:

Unavailability of medication is stressful because RHD patients always come to us stating that they could not find BPG medication. [N25].

Nurses emphasized the challenges in obtaining BPG and occasionally had to suggest that RHD patients speak with their prescribing physician about switching medication. In the absence of BPG, a nurse described conversation with the RHD patient as follows:

Unavailability of the medication is a problem. Even today, I have told one patient to buy from one pharmacy for this month, and it may not be available here in the next month. [N22].

In addition to the lack of medication, it was mentioned that patients can show up for routine follow-up without a prescription to receive an injection. One nurse response cites “*lack of a prescription”* as a reason why administering BPG is difficult.[N14].

#### Lack of reference guidelines

The significance of reference guidelines for self-updating on BPG medication and its administration, as well as for knowing the dosage, was explained by the nurses. According to nurses, reading and updating are essential. While it requires easily accessible reference guidelines, it is essential to understand the dosage, types of emergency drugs for shock treatment, and pain-reduction procedures while delivering BPG. The Lack of reference guidelines was discussed as:

Clear instructions on what to do and how to handle issues should be displayed on the injection room wall. [N1].

#### Limited Healthcare resource

Shortage of trained staff, uncomfortable injection rooms and limited availability of supplies were commonly discussed healthcare facilities-related physical opportunity barriers. According to majority of nurses interviewed, their BPG administration is being impacted by their workload. The proportion of staff assigned for injections and other services was not proportional. Nurses are compelled to perform several duties due to a severe staffing shortage. According to a nurse response:

It is difficult to administer safe injections due to a lack of organized setup, staffing shortages, and being busy. [N6].

Nearly all the nurses emphasized that to lessen their anxiety about BPG injections, the Ministry of Health and Healthcare facilities must increase their capability. Additionally, most of the participants highlighted the absence of a well-equipped and comfortable injection room affected their BPG administration practice. They also claimed that enough space should be available in the injection room to allow them to comfortably provide the injection and help them in the management of patient reaction to medication. However, an *“uncomfortable injection room with limited supplies”* [N24] remains a challenge.

Most of the nurses complained that their injection room was not comfortable and lacked the required supplies for proper administration of BPG. A nurse from the interviews included stated his injection room as:

The absence of a private, well-equipped injection room remains a significant issue. It is uncomfortable to provide BPG injections in this hospital’s injecting room. It can be difficult to maintain the privacy of patients. [N15].

### Social opportunity barriers

#### Rejection by RHD patients or community

BPG administration is still hampered by social variables such as patient rejection, patient perception of the drug, and poor staff communication. During the discussion, a nurse talked about how the rejection of RHD patients influenced the administration of BPG. “*Being rejected by RHD patients prevented me from giving this medication. Patients and attendants should have faith in you as a trustworthy provider; otherwise*,* they may believe that the negative effects of the medication are your fault.” [N11].* It has been discussed by many of the participants that patients may reject nurses because of the nature of the drug. Because BPG clots so quickly during injections, nurses may have to inject patients more than once, which could lead to frustration and a lack of trust in injection providers. Additionally, patients may refuse to take the prescription because they are distracted by rumors about it, which could result in the monthly BPG prophylaxis being rejected. Poor drug adherence is also a result of these social issues such as rumors in the community [N11, N15, N21].

#### Limited inter-professional collaboration and communication

Inter-professional collaboration or communication was discussed by nurses as another barrier to BPG administration. Nurses claimed there is limited communication between nurses and physicians prescribing the medication. A male nurse included in the interview stated his communication as:

Limited inter-professional communication. [N16].

Another study participant of this study stated inter-professional communication as: *“………there is no communication between the nurse and the physicians. There is nothing called communication. I don’t know the reason. I have never seen a nurse and physician collaborating to counsel a patient on BPG prophylaxis.”[N15].*

### Reflective motivation barriers

#### Limited intention to administer BPG

For a variety of reasons, almost all nurses are reluctant to administer BPG medication, even though it is a professional obligation. “The law is against the provider in case something happens” no one protects the in-charge nurse. [N10]. According to another nurse:

I don’t give injections to newly starting patients. I always ask them multiple times whether they are already familiar with the injection. [N24].

Nurses talked about the necessity of using media campaigning to improve BPG administration. Therefore, the task of altering public perception of this drug should fall to the Ministry of Health. It was said as follows in one of the interviews:

Media can reduce the fear of providers. The Ministry of Health should buy a little time to teach and influence the attitude of the patients and the providers. [N16].

Nurses raise conflicting issues regarding the intention to provide injections and the consequence of unintended outcomes of the medication. It is seen that a significant number of nurses have no intention problem. [N5, N10, N13,N17,N21,N23,N24]. However, they have doubts about when the patient’s life is in danger. Most of the injection providers nurses knew the drug was mandatory for RHD patients and had the intention to provide but the “what if” remains challenging. [N10, N17, N21 N23, N24].

#### Limited belief in capability/Lack of confidence to administer BPG

The nurses who participated in this study openly highlight the fact that confidence issues arise for a variety of reasons. The most frequently mentioned ones are lack of confidence on the part of prescribers, fear of side effects, absence of consent prior to injection, and inability to handle difficulties. The following statements about nurses’ confidence were made in the included interviews:

Nurses invite one another to give injections. I don’t believe nurses lack the necessary BPG administration knowledge, but I’m not sure if they have the confidence to inject and handle difficulties. [N3].

In addition to the providers’ lack of confidence, patients’ lack of trust in injectable providers continues to be a significant obstacle. *“The patient and attendant should trust you as a safe provider”*, one of the nurses said. [N11].

### Automatic motivation barriers

#### Fear of shock or collapse

Majority of the nurses who participated in this study reported that common impediments to administering BPG included fear of shock or collapse, legal issues, and other side effects. Nurses’ fear is also related to the lack of appropriate patient management equipment and supplies. The absence of the necessary equipment also contributes to the fear of administering BPG in case the patient is affected by drug adverse events. [N13, N17]. Another nurse also mentioned how the medication’s pain scares her and occasionally keeps her from providing it. [N23].

#### Fear of the law and medico-legal issues

Majority study participants comprehend the significance of administering BPG to patients with RHD. Fear of the law or medico-legal issues, however, continue to be major obstacles. Accountability was a hot topic in their answers and is still a big worry. The issue was expressed as follows:

The law does not let you go free if someone dies during the injection which prevents you from administering BPG injection. [N21].

#### Absence of informed consent

Nurses highly emphasized the need for informed written consent in the process of BPG administration. As the issues of accountability remain a key barrier, consented patients should get the injection. [N8]. One of the respondents stressed the importance of the provision of informed consent as:

Before receiving an injection, RHD patients who are taking BPG should give their informed consent. [N3].

## Discussion

This review identified different barriers and facilitators of BPG administration using an established behavioral model [called COM-B], which was not perviously reported. The COM-B model with the behavioral change wheel (BCW) will be used to discuss our findings in light of potential future intervention satrategies ( Supplement Table 2) [20].

Capability barriers such as limited skills in complication management, lack of training on safe BPG injections, misunderstandings, and lack of awareness of BPG prophylaxis were commonly discussed. Limited skills in BPG injection complication management remain a key capability barrier in BPG administration. This finding is supported by a systematic review [12] and studies conducted in Ethiopia [8, 15]. Our findings indicate that a lack of training on safe BPG injection is another reason for the failure to provide BPG injections, which aligns with another study conducted in Ethiopia [8]. These barriers could be addressed by the training and education intervention function of BCW. BCW’s training function could serve as a crucial intervention strategy for imparting safe injection skills. Training and education were also supported to increase confidence in dealing with BPG administration and management of complications. Additionally, training and education could clear medico-legal concerns of nurses related to BPG medication administration. Another important psychological capability barrier to BPG administration was misunderstanding of nurses about BPG prophylaxis and lack of awareness of BPG side effects and its guidelines. This finding is supported by an integrative review of studies conducted by Murray and colleagues [12]. The barriers to BPG administration by nurses could be addressed through further education and training. This recommendation is in line with a systematic review of studies on nursing practice [12] and other studies [1, 9].

Identified opportunity barriers for BPG administration were unavailability of medication guidelines, limited healthcare capability, absence of a comfortable injection room, rejection by RHD patients, and ineffective communication. This finding showed that access challenges to BPG and other emergency medications continue to be a major barrier to BPG delivery. Nurses emphasized that the delivery of BPG was significantly impacted by the lack of emergency medications. The overall unavailability of necessary medications continues to be a source of stress and a significant barrier, leading to occasional discussions about switching to alternative medications. There were times when patients also presented without medication and is supported by a study conducted by Pan-African Society of Cardiology Task Force on RHD [4] and a study from Ethiopia [8]. This barrier could be resolved by using the environmental restructuring and enablement function of BCW. This recommendation is in line with a study conducted in Zambia especially with increasing access and training [9].

The inaccessibility of reference guidelines remains a physical opportunity barrier for the provision of BPG prophylaxis. It impacted self-updating needs on both the medication and its adverse events management. The finding is supported by a study conducted by Pan-African Society of Cardiology Task Force on RHD [4]. The barrier can be solved by environmental restructuring, such as adding an object [in this case, ensuring the availability of reference guidelines]to existing environment/hospital or healthcare facilities. Our findings showed limited healthcare capacity such as a shortage of trained staff and the absence of a well-equipped and comfortable injection room affected their BPG administration practice. Regarding the lack of availability of well-equipped injection room is supported by investigators in Uganda [10]. These factors could be improved through training, environmental restructurings, and an enablement intervention function of BCW. Environmental restructuring, such as making the injection room adequately spaced, comfortable, and equipped with required supplies could be one solution in addition to assigning an adequate number of trained BPG injection providers. Enablement function of the BCW intervention could also be considered in making the environment conducive and ensuring enough space in the injection room to allow injection providers to comfortably provide the injection and help them in the management of patient reaction to medication.

BPG administration is still hampered by social variables such patient rejection, patient perception of the drug, and poor staff communication. Being rejected by RHD patients remains a social opportunity barrier for BPG administration. In this study, there was limited interprofessional collaboration and communication between nurses and prescribing physicians. The finding supplements a report from a systematic review conducted on nurses practice on BPG administration [12] and a qualitative study conducted in Uganda [10]. Moreover, the patients could reject the nurses due to the nature of the drug and related rumors of side effects such as clots. These factors could be addressed by education, training and the persuasion function of BCW. Patient counseling or education on the drug side effects and related rumors of adverse events could help in reducing rejection. Communication barriers should be addressed through the training of health care providers. This recommendation is agreement with a systematic review conducted on nurses [12].

Motivational barriers identified in this study were lack of intention to provide BPG, intention to give BPG vs. consequence of the medication, lack of confidence, fear of shock, and law. In this study, limited intention to administer BPG was reported for a variety of reasons, almost all nurses were reluctant to administer BPG, even though it is their professional obligation to do so but claiming that the law is against if an adverse event occurs which is supported by a study conducted by Pan-African Society of Cardiology Task Force on RHD [4]. However, there were conflicting issues raised by nurses regarding the intention to provide BPG versus the consequences related to BPG injections. Furthermore, the nurses also called upon the need for a media campaign by the Ministry of Health to improve perceptions towards BPG prophylaxis in both patients and healthcare providers, especially nurses.

Lack of confidence as a result of perceived BPG adverse events, lack of confidence from prescribers, lack of consenting process before BPG injection and perceived inability to handle difficulties remain major challenges for BPG administration and is in agreement with a study conducted by Pan-African Society of Cardiology Task Force on RHD [4]. Another reason for the lack of confidence is related to patients’ lack of trust in providers that continues to be a significant obstacle. These reflective motivational barriers such as limited intention, intention vs. consequence, and limited confidence could be addressed by education, training, and persuasion intervention functions of BCW. In this study, fear of shock/collapse, the law, and an absence of informed consent were automatic motivational barriers to BPG administration. In our study, the most common impediments to administering BPG included fear of shock or collapse, legal issues, and other side effects and related lack of appropriate patient management equipment and supplies. Fear of accountability or medico-legal issues continues to be a major obstacle. Issues of accountability remain a key barrier in BPG provision due to the absence of informed consent in the process of BPG administration to reduce the fear of nurses. It is highly recommended from the side of nurses that consented patients should get the injection. This finding is consistent with a study conducted by Ali and colleagues [4]. These all-automatic motivational barriers could be addressed by the education, training, persuasion, and enablement intervention functions of BCW.

### Strength and limitations of the study

This study has several strengths. First, it is the first qualitative study that used a comprehensive behavior change model, COM-B, to assess BPG administration experiences of nurses. Second, this study also pointed out possible intervention areas for the identified barriers helping nurses and healthcare system leadership to address these challenges. Third, the study was conducted in the national RHD referral center and included nurses with variable levels of experience to understand their BPG administration experiences. Limitations of the study were also present. The research was conducted in a single center referral center, which may limit transferability to non-academic referral centers and in other geographic locations in Ethiopia where nurses may be even more reluctant to administer BPG. Nurses at the referral center where the study was conducted may be more educated about BPG administration for RHD which may also limit generalizability to other settings. There were also designated cardiology clinics and masters degree prepared cardiovascular nurses who may be more aware of BPG administration for RHD than nurses in other geographic locations. Referral centers also have greater resources for training nurses, providing ongoing in-service sessions, ensuring availability of emergency medication and using evidence-based guidelines for administering BPG compared to other healthcare facilities in Ethiopia.

## Implications for practice and research

The findings from this study can be used to design an implementation strategy to improve BPG prophylaxis administration. The study identified different barriers across the three domains of COM-B model which could be addressed by using BCW intervention functions and behavioral change techniques. Nurses’ capability barriers limited skills in safe injection and related management, misunderstandings and lack of awareness of BPG prophylaxis could be addressed by the provision of skills training and a short course on BPG prophylaxis. Healthcare managers should pay attention to address the unavailability of medication, reference guidelines and limited trained providers. Whenever possible RHD patients’ concerns should be addressed and related BPG rumors of adverse events a poor outcome explained. BPG prescribers and injection providers should establish a positive communication environment. Regular education and training should be given to improve understanding of and clear misconceptions about BPG prophylaxis. Safe BPG administration procedure protocols and adequately stocked injection provision rooms need to be provided. Addressing capability, opportunity and motivational barriers as described in this paper should form the basis of a multi-component strategy that is urgently needed to reduce morbidity and mortality of RHD patients.

## Conclusions

This study guided by the COM-B and BCW documented a range of barriers and facilitators of BPG administration. Poor nurses’ intention to provide BPG for RHD patients included fear of adverse events, medico-legal concerns, absence of consent, absence of comfortable BPG injection rooms and emergency medication to treat possible BPG adverse reactions. Education, environmental restructuring, training, persuasion (effective communication) and enablement are potential strategies that may be effective in increasing nurses administer BPG for RHD patients. Large scale research studies are also recommended to thorougly understand factors then identify contextual strategies to address barriers and capitalize on facilitators. Finally, future studies are also recommended to test the strategies that will be developed based on the current study findings.

## Supplementary Information

Below is the link to the electronic supplementary material.

Supplementary Material 1

## Data Availability

The datasets used during the current study are available from the corresponding author on reasonable request.
