# Supplementary material for: Exploring nurses’ experience with benzathine penicillin administration for patients with rheumatic heart disease in Ethiopia: a qualitative study
Source: BMC Nurs. 2026 Aug 7;25:1069. doi: 10.1186/s12912-026-05197-4 (PMC13737516; doi:10.1186/s12912-026-05197-4)
Supplement: Supplementary file 1 [file 12912_2026_5197_MOESM1_ESM.docx]

**Supplement Table 1: In-depth interview guides**

| **COM-B domain** | **#** | **Interview guide** |
| --- | --- | --- |
| Capability: Physical | 1 | Age |
|  | 2 | Gender 1. Male 2. Female |
| Capability: Psychological | 3 | Educational qualification |
| Opportunity: physical | 4 | Year of experience |
| Capability: Psychological | **5** | Let us start with general information about your education/training. |
| Physical capability/  Reflective motivation  Automatic motivation | **6** | What is your experience of working with RHD patients? (Probes: follow up of RHD patients, frequency of seeing patients etc.) |
|  | **7** | What does your experience of BPG administration look like? (**Probes**: confidence in providing BPG, challenges such as experience of side effects, RHD severity etc.?) |
| Psychological capability/physical opportunity | **10** | Can you tell me what must be carried out during BPG administration?  **Probes:** check allergies, prepare items needed, prepare the injection, prepare the patient, and administer the injection and observe the patient. |
| Reflective motivation | **11** | How do you describe your intention of providing BPG prophylaxis to your RHD pts? **Probe**: concerns such as fear of safety, side effects etc.; belief, perceptions |
| Reflective motivation/Physical opportunity | **12** | In your opinion, what factors do affect your BPG administration practice? **Probe:** Nurse related: knowledge, training, lack of awareness of safe injection, other social factors. Healthcare capability: drug availability, staffing, injection room. Patient related. |
| Reflective motivation | **13** | What do you think are the consequences of poor BPG prophylaxis adherence? |
| Reflective motivation  Social opportunity | **14** | How do you see your professional commitment in supporting RHD patients to take BPG prophylaxis and adhere to their BPG prophylaxis?  **Probing**: communication among treating staff/inter-professional collaboration in BPG prophylaxis |
| Motivation reflective/automatic | **15** | How do you motivate your RHD patients to improve their BPG prophylaxis adherence? Probe: Counseling patients, referral, linkage. Patients’ characteristics with many problems due to gender, residence, clinical status? |
| Opportunity: Physical | **16** | What ways of working will help your BPG administration experience for your RHD patients? |
| NA | **17** | What additional experience would you like to share with us? |

Our discussion was very helpful. Thank you very much for talking with me today.

**Stop the record!**

**Supplement Table 2: Definition of the BCW intervention functions**

| BCW Interventions | Definitions | Examples |
| --- | --- | --- |
| Education | Increase knowledge or understanding | Providing information to promote BPG administration by nurses and BPG uptake by RHD patients. |
| Persuasion | Using communication to induce positive or negative feelings or stimulate action | Using imagery to motivate increase BPG administration |
| Training | imparting skills | Provide training to reduce misunderstanding about BPG and then to increase BPG administration skills. |
| Environmental restructuring | Changing the physical or social context | Ensure availability of medication, reference guidelines, and privately equipped injection room with all needed supplies, etc. |
| Enablement | Increasing means/reducing barriers to increase capability or opportunity.  (Capability beyond education and training; opportunity beyond environmental restructuring). | Behavioral support of nurses in enabling BPG administration, subsidizes BPG and other emergency medication, establishing comfortable injection rooms etc. |
| Incentivization | Creating expectations of reward | NA |
| Coercion | Creating expectations of punishment or cost |  |
| Restriction | Using rules to reduce the opportunity to engage in target behavior (or to increase the target behavior by reducing the opportunity to engage in competing behaviors) | NA |
| Modelling | Providing an example for people to aspire to or imitate | NA. |
